# RNA m^6^A modification: Mapping methods, roles, and mechanisms in acute myeloid leukemia

**DOI:** 10.1097/BS9.0000000000000131

**Published:** 2022-07-20

**Authors:** Rong Yin, Yashu Li, Wen Tian, Fuling Zhou, Haojian Zhang

**Affiliations:** a Department of Hematology, Zhongnan Hospital, Wuhan University, Wuhan, People’s Republic of China; b Frontier Science Center for Immunology and Metabolism, Medical Research Institute, Wuhan University, Wuhan, People’s Republic of China

**Keywords:** Acute myeloid leukemia, Mapping, RNA m^6^A modification

## Abstract

N^6^-Methyladenosine (m^6^A) is the most abundant modification in eukaryotic mRNA, and plays important biological functions via regulating RNA fate determination. Recent studies have shown that m^6^A modification plays a key role in hematologic malignancies, including acute myeloid leukemia. The current growth of epitranscriptomic research mainly benefits from technological progress in detecting RNA m^6^A modification in a transcriptome-wide manner. In this review, we first briefly summarize the latest advances in RNA m^6^A biology by focusing on writers, readers, and erasers of m^6^A modification, and describe the development of high-throughput methods for RNA m^6^A mapping. We further discuss the important roles of m^6^A modifiers in acute myeloid leukemia, and highlight the identification of potential inhibitors for AML treatment by targeting of m^6^A modifiers. Overall, this review provides a comprehensive summary of RNA m^6^A biology in acute myeloid leukemia.

## 1. INTRODUCTION

Maintenance of hematopoietic system throughout the lifetime relies on hematopoietic stem cells (HSCs), which possess unique self-renewal capacity and multilineage differentiation potential and replenish all lineages of blood and immune cells.^1^ The imbalance of hematopoietic homeostasis can lead to the development of various blood diseases such as leukemia, bone marrow failure, myelodysplastic syndrome. Genetic or epigenetic alterations occurred in hematopoietic stem/progenitor cells (HSPCs) transforms them into leukemia stem cells (LSCs), which subsequently initiate the development of leukemia, such as acute myeloid leukemia (AML).^2^ AML is an aggressive and fatal hematologic malignancy, which is characterized by uncontrolled expansion and impaired differentiation of myeloid progenitor cells.^3^ In clinic, chemotherapy is still the standard treatment strategy for most newly diagnosed patients with AML. Currently, the 5-year survival rate is around ~30% for AML patients under 60 years old.^4,5^ Thus, exploring the molecular mechanisms of AML development and investigating potential therapeutic targets for AML treatment remain a big challenge in this field.

Leukemogenesis is a multistage process that is governed by complex regulatory networks.^6^ Normally, HSCs predominantly reside in a quiescence state that is coupled with different controlled systems such as metabolism, protein synthesis and stress response system.^7–9^ Dysregulation in these tight and dynamic controls of HSC activity results in malignant transformation. Increasing evidence has suggested that mutations of some epigenetic modifiers such as DNMT3A and TET2 are common early events of leukemogeneis.^10,11^ These founder mutations result in epigenetic alterations and aberrant transcriptional networks, which subsequently prime HSPCs into a state called pre-leukemic stem cells (pre-LSCs). Secondary or further genetic alterations will fully transform these pre-LSCs into LSCs.^12^ Histone modification is another aspect of epigenetic regulation. For instance, translocations of *MLL*, encoding a histone methyltransferase, are recurrently found in chromosomal rearrangements involving 11q23 in AML, highlighting the significance of this epigenetic modifying enzyme in AML development.^13^ Other histone modification enzymes, such as DOT1L, EZH2, ASXL1, KDM1A, also involve in leukemogenesis by providing a selective advantage to LSCs.^14–17^

Similar to DNA methylation and histone modification, RNA modification has recently been implicated in AML initiation and progression. N^6^-methyladenosine (m^6^A) is the most enriched modification in eukaryotic mRNA and long non-coding RNA, and participates in regulating RNA metabolism by affecting RNA splicing, nuclear export, translation, and degradation.^18^ Increasing evidence suggests that RNA m^6^A modification acts as an important adaptive-response mechanism in regulating various biological processes at the epitranscriptomic level. Recent studies have also shown that m^6^A modification plays an important role in many stages of normal and malignant hematopoiesis. These findings not only uncover a new regulatory layer in leukemogenesis, also provide novel and promising therapeutic strategies for targeting leukemia. Thus, in this review, we first describe m^6^A machinery, and then focus on AML and discuss recent advances of RNA m^6^A modification in AML.

## 2. RNA m^6^A MODIFICATION

**Figure 1.**
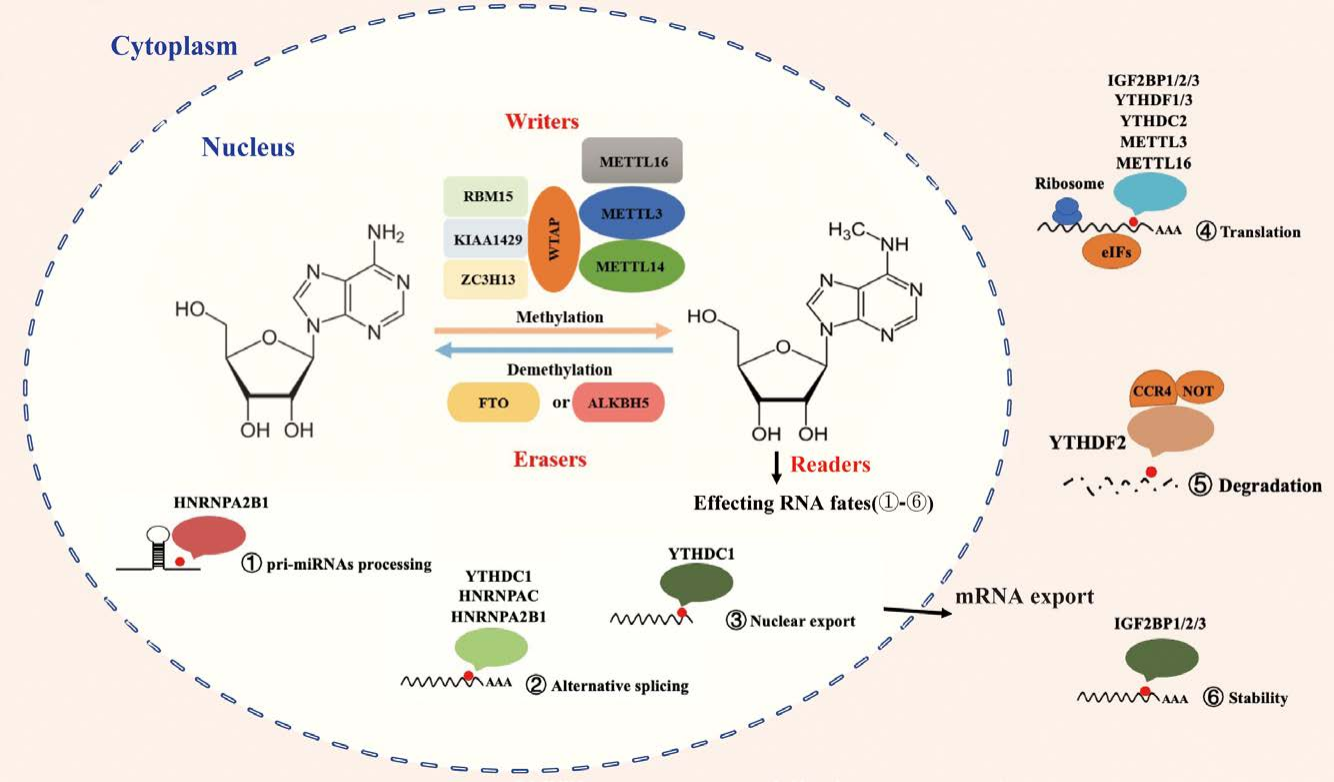
m^6^A modification in RNA metabolism. m^6^A modification is reversible and dynamically regulated by methyltransferase complex (including METTL13, METTL14, METTL16, WTAP, RBM15, KIAA149, and ZC3H13) and demethylase including ALKBH5 and FTO. RNA binding proteins (including YTHDC1/2, YTHDF1-3, IGF2BP1-3, HNRNPA2B1, and HNRNPC) participates in regulating various stages of RNA metabolism including alternative splicing, nuclear export, pri-miRNA processing, translation, stability, and degradation.

Among over 170 different chemical modifications of RNA that have been identified to date, m^6^A is the most common one in mammalian mRNA. Comprehensive analysis of mRNA m^6^A using by establishing high-throughput sequencing reveals enrichment of m^6^A sites nearby the 5′ untranslated regions (5′-UTR), stop codon, 3′ untranslated regions (3′-UTR), and long internal exons with conserved RRACH motifs (R = G, A, and U; H = U, A, and C).^19^ Each transcript contains 3 to 5 or more m^6^A modification sites, accounting for 0.1% to 0.4% of total adenine (m^6^A/A).^20^ It is known that m^6^A modification is reversible and dynamically regulated by methyltransferase complex that catalyzes the installation of m^6^A, and by demethylases responsible for m^6^A removal. The methyltransferase complex, also called writer, is a multicomponent nuclear complex composed of 2 methyltransferase-like proteins (METTL3 and METTL14) and several regulatory proteins (WTAP, VIRMA, CBLL1, RBM15/RBM15B, METTL16, and ZC3H13). m^6^A modification is removed by either ALKBH5 or FTO (also called eraser), both of which belong to the ALKB family of the e(II) and α-ketoglutarate-dependent dioxygenases. In general, m^6^A methylation sites can be recognized by some specific RNA binding proteins, including YTHDC1/2, YTHDF1-3, and IGF2BP1-3, which play different roles in fine-tuning mRNA metabolism. As these RNA m^6^A machineries have been widely discussed in many decent literatures, we just briefly describe them below (Fig. 1).

### 2.1. RNA m^6^A methyltransferases

METTL3 and METTL14 compose the core of the methyltransferase complex, and METTL3 is currently the sole catalytic component in this complex while METTL14 plays an important role in structural stabilization and RNA-substrate binding. This complex catalyzes the transfer of methyl group from S-adenosylmethionine (SAM) to the sixth N atom of RNA adenosine. METTL3 has the leading helix structure (LH) and nuclear localization signal (NLS) domains, which mediate the interaction of METTL3 with METTL14.^21–23^ CCH-type zinc finger domain (ZFD) of METTL3 serves as the target recognition domain and fulfills the methyltransferase activity of this complex.^24^ Moreover, both METTL3 and METTL14 have SAM structure binding domain—methyltransferase domain (MTD).

The METTL3–METTL14 complex composes several key subunits that guides the core complex onto specific mRNA regions and are responsible for determining its activity and specificity. WTAP recruits METTL3 and METTL14 to nuclear speckles for m^6^A methylation.^25^ VIRMA (KIAA1429) is identified as a scaffold that orchestrates the catalytic core components to guide region-selective methylation. RBM15 is recruited to the methyltransferase complex to promotes RNA methylation.^26^ ZC3H13 mainly functions to retain the ZC3H13–RBM15–WTAP complex in the nucleus to regulate m^6^A methylation.^27,28^ RBM15, a homolog of spen, binds and recruits the m^6^A methyltransferase complexes to XIST, leading to m^6^A formation that mediates transcriptional repression.^29^ VIRMA contains the RBP domain, and facilitates m^6^A writer complex to the 3′UTR region.^30^ Post-translational modifications can also affect the activity of METTL3. For instance, TBK1 is a key kinase of antiviral pathways, and a recent study show that TBK1 directly phosphorylates METTL3 at serine 67 and promotes its activation and m^6^A modification to stabilize *IRF3* mRNA.^31^

Besides METTL3-METTL14 complex, METTL16 is identified as an alternative m^6^A methyltransferase. Human MAT2A encodes the SAM synthetase expressed in most cells. Recent study found that METTL16 functions as a m^6^A methyltransferase and catalyzes the methylation of MAT2A hairpin HP1, which regulates MAT2A expression by affecting its intron splicing. Due to the similarity between HP1 and U6 snRNA methylation sites, METTL16 is also responsible for m^6^A methylation of U6 snRNA.^32^ METTL16 can also deposit m^6^A mark on the pre-mRNA. Recent work found that the C. elegans writer METT-10 (the orthology of mammalian METTL16) installs an m^6^A mark on the 3′ slice site of SAM synthetase pre-mRNA, which inhibits its proper slicing and protein production.^33^ Splicing inhibition by 3′ splice site m^6^A is conserved in mammals. METTL16 also binds to other ncRNA as well as numerous lncRNA, suggesting that it might be responsible for m^6^A methylation of these RNAs.^34^ In sum, additional m^6^A methyltransferases remain to be identified.

### 2.2. RNA m^6^A demethylases

FTO and ALKBH5 are known as the 2 m^6^A demethylases, and both belong to the ALKB family. FTO was widely studied as a fat mass and obesity-associated protein associated to metabolic disorders such as diabetes and obesity. Recently, FTO was reported to have efficient oxidative demethylation activity and can sequentially oxidize m^6^A to hm6A or fm6A, which are unstable and can be hydrolyzed to adenine.^35^ FTO can also catalyze the demethylation of m^6^Am on mRNA and snRNAs, and m^1^A on tRNA. The cellular distribution of FTO affects its access to different RNA substrates, which might play a role in determining FTO’s substrate specificity. FTO in the nucleus has a higher affinity for m^6^A, while FTO in the cytoplasm has a higher affinity for m^6^Am.^36^ Currently, factors that influence the location of FTO remain unknown yet. ALKBH5 is another demethylated enzyme that specifically recognizes RNA m^6^A and directly remove RNA m^6^A.^37^ Although α-KG and Fe2+ are essential for the demethylation activity of FTO and ALKBH5, it remains unclear how the activities and specificity of these m^6^A erasers are regulated in different contexts.

### 2.3. RNA m^6^A readers

RNA m^6^A readers recognize different m^6^A sites under distinct contexts and function as the key in regulating the fates of m^6^A targets. One group of m^6^A readers contain the YT521-B homology (YTH) domain that is responsible for recognizing m^6^A, including YTH domain family 1-3 (YTHDF1-3) and YTH domain containing 1-2 (YTHDC1/2). Even belonging to the same class, these readers mediate different fates of m^6^A-modified mRNAs. YTHDF1 facilitates translation initiation of m^6^A-modified mRNAs by interacting with eukaryotic initiation factor 3,^38^ while YTHDF2 mainly destabilizes m^6^A-modified mRNAs via directly recruiting the CCR4–NOT deadenylase complex in the cytoplasm.^39^ YTHDF3 facilitates mRNA translational efficiencies of its targets and is dependent on m^6^A methylation. In addition, a recent study has reported that YTHDF3 cooperates with YTHDF1 to facilitate translation of protein synthesis and affects YTHDF1-mediated decay of methylated mRNA.^40^ YTHDC1 mainly locates in the nucleus and regulates the export of m^6^A-tagged mRNAs from nucleus to cytoplasm.^41–44^ YTHDC1 also mediates mRNA splicing by recruiting 2 competitive mRNA splicing factors serine/arginine-rich splicing factor 3 (SRSF3) and SRSF10.^44^ YTHDC2 is present both in the cytoplasm and nucleus, where it affects translation and stability of its target mRNAs.^45^

Another group of m^6^A readers is IGF2BP1-3, which have 56% identify on their amino acid sequence homology. These readers contain 4 repetitive KH domains, and the third and fourth KH domains are responsible for recognizing m^6^A sites. IGF2BPs predominantly enhance the stability and translation of m^6^A-modified mRNAs.^46^ IGF2BP3 is also required for pre-mRNA splicing.^47^ Our recent work reveals that YBX1 cooperates with IGF2BPs to promote the stability of m^6^A-tagged transcripts,^48^ suggesting YBX1 is 1 component of IGF2BP regulatory machinery. Several heterogeneous nuclear ribonucleoproteins (HNRNPs) including HNRNPC, HNRNPG, and HNRNPA2/B1 also function as m^6^A reader. HNRNAPC as a nuclear protein can regulate alternative splicing of m^6^A-modified transcripts. HNRNPA2B1 functions as a m^6^A nuclear modification reader that can regulate primary microRNA processing and alternative splicing.^49^ 5′UTR m^6^A modification also can be recognized by HNRNPG and YTHDC1, which prevent the integrator complex from splicing the nascent RNA and promotes the transcription of the nascent RNA.^50^ Overall, m^6^A readers are crucial in determining the fate of their target mRNAs via recruiting diverse regulatory machinery to m^6^A sites.

## 3. TECHNOLOGIES FOR MAPPING m^6^A

**Figure 2.**
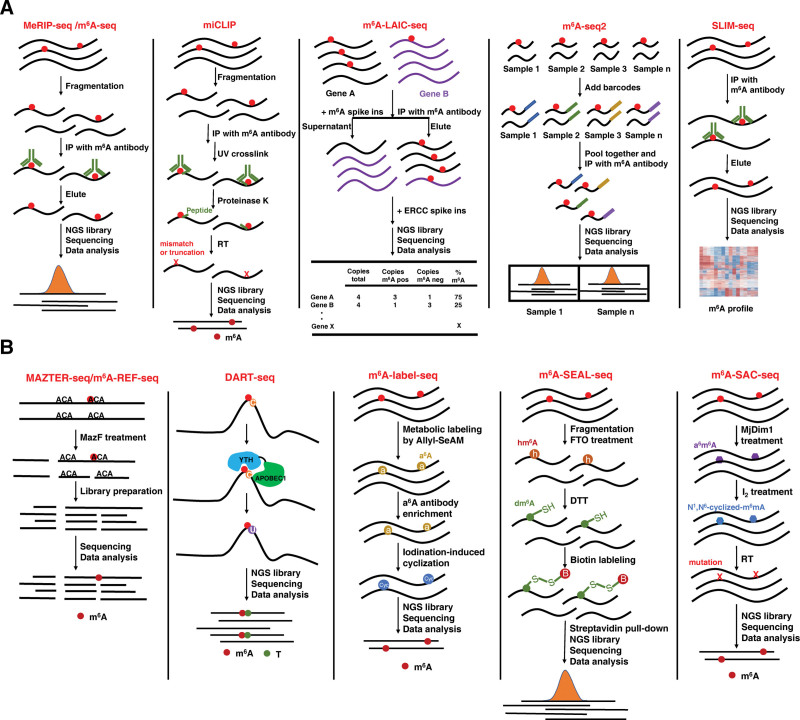
Outlines of different strategies for m^6^A mapping. (A) Antibody-dependent mapping methods. MeRIP-seq/m^6^A-seq fragment the RNA to ~100 nt sized fragments following with immunoprecipitation with anti-m^6^At antibody to enrich RNA fragments containing m^6^A. miCLIP detect m^6^A positions at single-nucleotide resolution by inducing mutation or truncation. m^6^A LAIC-seq using in vitro transcripted m^6^A spike-ins to quantify methylated versus nonmethylated transcripts. m^6^A-seq2 add RNA barcodes to fragmented RNA originating from distinct samples, and pooled together followed by a single m^6^A-IP performed on pooled RNA samples. SLIM-seq combining the advantages of m^6^A-LAIC-seq and SMART-seq, decoding m^6^A modification on full-length transcript with high sensitivity for low-input. (B) Antibody-independent mapping methods. MAZTER-seq/m^6^A-REF-seq use MazF to cleave RNA at unmethylated sites occurring at ACA motifs. DART-seq use YTH domain to recognize m^6^A and APOBEC1 to mark m^6^A site. m^6^A-label-seq metabolically incorporate allyl-group into the adenosine sites (a^6^A) then transformed (a^6^A) into N^1^, N^6^-cyclized adenosine (cyc-A), the cyc-A induced misincorporation during reverse transcription. m^6^A-SEAL-seq use FTO’s enzymatic oxidation and a DTT-mediated thiol-addition reaction to convert m^6^A into dm^6^A, then labelled by biotin and pulled down by streptavidin. m^6^A-SAC-seq used Dim1/KsgA family of dimethyltransferases, converting m^6^A into *N*^6^,*N*^6^-dimethyladenosine (m^6^_2_A) in consecutive methylation reactions.

The field of epitranscriptomics was beginning to revive around 10 years ago, which mostly benefit from the seminal studies that establish a strategy for mapping the transcriptome-wide m^6^A profiling.^19,51^ These works have ignited the exponential research about RNA modifications under various physiological and pathological conditions, and the growth of epitranscriptomic research also stimulated the development of new tools or approaches for detecting and mapping RNA marks even in lower-abundance RNA species. Hereby, we briefly discuss the current methods for mapping the transcriptome-wide m^6^A modification (Table 1). Based on the strategies for detecting or recognizing m^6^A sites, these methods can be simply divided into 2 groups: antibody-dependent mapping and antibody-independent mapping (Fig. 2).

**Table 1:** A summary of advantages and limitations of m^6^A mapping methods.

m^6^A detection methods	Advantages	Limitations	Ref
MeRIP-seq/m^6^A-seq	First method for global view of m^6^A	Large amounts of starting RNA Low resolution	^51^
miCLIP-seq	Single-nucleotide resolution	Large amounts of input RNA Non-specific binding of antibody	^52^
m^6^A-LAIC-seq	Quantify m^6^A stoichiometry	Low resolution	^53^
m^6^A-seq2	Allows quantification across genes and samples	Low resolution	^54^
SLIM-seq	Low input material High sensitivity	Low resolution	^55^
MAZTER-seq/m^6^A-REF-seq	Single-nucleotide resolution Quantitative tracking of m^6^A	Only detect 16%–25% of m^6^A sites	^56^
DART-seq	Allow detect m^6^A accumulation Low amounts of input RNA	Low sensitivity Only identify partial m^6^A signal	^57^
m6A-label-seq	Single-nucleotide resolution	Low efficiency Lack stoichiometric information	^58^
m^6^A-SEAL-seq	Less false-positive signals	Lack stoichiometric information Unstable efficiency	^59^
m^6^A-SAC-seq	Single-nucleotide resolution Quantitative tracking of m^6^A	Low specificity and efficiency	^60^

### 3.1. Antibody-dependent m^6^A mapping

The first high-throughput method for mapping a transcriptome-wide m^6^A landscape was developed independently by 2 groups in 2012.^19,51^ Based on the high sensitivity and selectivity of anti-m^6^A antibody in detecting m^6^A, researchers from these 2 groups established the same strategy that combines m^6^A-specific methylated RNA immunoprecipitation with next-generation sequencing, thus this method is called MeRIP-seq or m^6^A-seq. The procedure involves randomly fragmenting the RNA to ~100 nt sized fragments following with immunoprecipitation with anti-m^6^A antibody to enrich RNA fragments containing m^6^A. This method first identifies more than 7000 mammalian genes that contain m^6^A in their transcripts, and reveal the distribution of m^6^A sites that are enriched near stop codons and in 3′ UTRs. These seminal studies provide a tool for investigating m^6^A profiling in a transcriptome-wide manner, and also indicate that RNA m^6^A has a fundamental role in regulating gene expression. However, the approach has several limitations, including low resolution and a requirement of large amounts of starting RNA. Since then, different mapping tools or strategies have been developed to reach more effective and accurate m^6^A profilings.

Although m^6^A positions could be predicted from MeRIP-seq by searching for DRACH motifs near the point of highest read coverage, precisely identifying m^6^A residues at high resolution is challenging. Due to the size of fragmented RNA, precise m^6^A positions cannot be identified. Considering that cross-linked proteins to RNA in living cells would result in mutations or truncations in the cDNA when it is reverse transcribed, researchers introduce UV cross-linking and immunoprecipitation for m^6^A antibody-enriched RNA fragments, named miCLIP.^52^ miCLIP enables to detect m^6^A positions at single-nucleotide resolution, and can also detect m^6^Am, a modification found at the first nucleotide of certain mRNAs. miCLIP requires large amounts of input materials. In addition, different commercial m^6^A antibodies might cause distinct mutation or truncation patterns in the cDNA, which would bring a lot of difficulties and noises for decoding the m^6^A residues.

To quantitatively deconvolute methylated to nonmethylated transcripts in a transcriptome-wide manner, m^6^A-level and isoform-characterization sequencing (m^6^A-LAIC-seq) was developed. This approach does not fragment the RNA before anti-m^6^A RNA immunoprecipitation, instead simultaneously sequences intact full-length transcripts in both m^6^A-positive and m^6^A-negative fractions following RNA immunoprecipitation. Importantly, in vitro transcripted m^6^A spike-ins are introduced to quantify methylated versus nonmethylated transcripts.^53^ Thus, this method allows to determine the methylation stoichiometry of different transcript isoforms at the full-length levels rather than the m^6^A position levels.

One of the major limitations of these above methods are the need for high input amounts. To overcome this limitation, 2 new approaches (m^6^A-seq2 and SLIM-seq) were recently developed. In m^6^A-seq2, barcoded RNA adapters are added to fragmented RNA originating from distinct samples, and pooled together followed by a single m^6^A-IP performed on pooled RNA samples instead of on a single sample. Thus, this multiplexed m^6^A-seq2 reduces the requirements of input materials, and also reducing technical variability which enables quantification of m^6^A levels across genes and samples.^54^ Recently, in order to explore m^6^A landscape of rare cell populations, we developed a highly sensitive and efficient super-low-input m^6^A sequence (SLIM-seq) by combining the advantages of m^6^A-LAIC-seq and SMART-seq.^55^ SLIM-seq focuses on decoding m^6^A modification on full-length transcript at the expense of the regional information of m^6^A peaks. Using in vitro-synthesized transcript *Luciferase* without m^6^A and *GFP* containing less than 0.5% m^6^A-modified adenosine that mimics the physiological level of m^6^A abundance, we confirm the consistent and comparable high efficiency of this strategy for different amounts of input, as few as 10 ng transcripts. Thus, a strong concordance was also observed using 5000 primary hematopoietic progenitor cells. Importantly, the inherent non-specific binding issue of m^6^A antibody is also taking into account in SLIM-seq, and adaptions in this approach significantly increase the specificity of SLIM-seq. Thus, SLIM-seq provides us a strategy to map transcriptome-wide m^6^A-tagged mRNAs for rare cells. As mentioned above, this method cannot provide the regional information of m^6^A peaks. SLIM-seq intends to decode m^6^A modification of whole transcript, which endows this strategy with high sensitivity for low-input. Notably, m^6^A largely determines mRNA abundance by regulating RNA decay and stability, its consequence on mRNA fate can be inferred by integrating expression data.

### 3.2. Antibody-independent m^6^A mapping

Antibody-based approach has some limitations including cross-reactivity to other RNA modifications, and limited utility for quantification of m^6^A stoichiometry. This raises several mapping strategies that are independent on m^6^A antibody. MAZTER-seq relies on the ability of the bacterial RNase MazF to cleave RNA at unmethylated sites occurring at ACA motifs, but not at the methylated counterparts m^6^A-CA.^56^ MAZTER-seq is able to systematically quantify m^6^A signal at single-nucleotide resolution. A same strategy was also applied in m^6^A-REF-seq.^61^ However, due to differential cleavage of by an RNase, this method only detects a small portion (16%–25%) of m^6^A sites.

APOBEC1 is a cytosine deaminase that targets DNA and RNA to induce cytosine-to-uracil (C-to-U) editing. By fusing APOBEC1 to the m^6^A-binding YTH domain of YTHDF2, m^6^A-adjacent cytidines could be induced to uracil by APOBEC1-YTH, which are detected using standard RNA-seq.^57^ This approach is named DART-seq (deamination adjacent to RNA modification targets), and is antibody-independent. DART-seq can detect about 79% of the edited mRNAs even with as little as 10 ng of total RNA as input. However, this approach only detects partial m^6^A signal that is recognized by YTH. In addition, the efficiency of APOBEC1-YTH in induing C-to-U transition in vivo or in vitro also need to be considered, and the specificity of YTH domain may also bring some biases.

Although the inert chemical property of RNA m^6^A poses a big obstacle to detect, many efforts attempt to alter this feature of m^6^A in developing antibody-free chemical approaches for m^6^A mapping. Recently, a metabolic labeling method, m^6^A-label-seq, is established to map m^6^A transcriptome-wide at base resolution. m^6^A-label-seq feeds cells with precursors of allyl-SeAM, Se-allyl-l-selenohomocysteine, to metabolically incorporate allyl-group into the adenosine sites (a^6^A) that are supposed to be m^6^A-modified.^62^ After enrichment with a^6^A antibody, a^6^A signals were transformed into N^1^, N^6^-cyclized adenosine (cyc-A) using the iodination-induced cyclization reaction. During reverse transcription, the cyc-A induced misincorporation. Therefore, the site can be detected through NGS. m^6^A-label-seq only can be used in cells and relies on the enrichment of a^6^A antibody. The number of identified m^6^A sites is limited, possibly due to the RNA a^6^A labeling yield. The incorporation efficiency of a^6^A need to be further improved. Considering that FTO oxidize m^6^A to hm^6^A in 5 minutes and then slowly oxidizes this hm^6^A to N^6^-formyladenosine, another strategy, m^6^A-SEAL, couples FTO’s enzymatic oxidation of m^6^A to hm^6^A with a DTT-mediated thiol-addition reaction to convert unstable hm^6^A into the more stable N6-dithiolsitolmethyladenosine (dm^6^A). These dm^6^A-marked RNA can be labeled by biotin and pulled down by streptavidin.^59^ Similarly, the oxidation efficiency of FTO need to be considered when using this method. In addition, both m^6^A-label-seq and m^6^A-SEAL lack stoichiometric information.

To detect and quantify m^6^A levels across the transcriptome at single-nucleotide resolution, m^6^A-SAC-seq is recently established. This method used the Dim1/KsgA family of dimethyltransferases, which transfer the methyl group from SAM to adenosines, converting m^6^A into *N*^6^,*N*^6^-dimethyladenosine (m^6^_2_A) in consecutive methylation reactions.^60^ This method needs a low abundance of input materials (about ~30 ng of poly(A) or rRNA-depleted RNA), and can provide stoichiometric information of m^6^A modification. Although it seems that m^6^A-SAC-seq could overcome the current technological bottleneck for m^6^A mapping, the specificity and efficiency of Dim1 remains an important issue.

All of these main methods discussed above have their advantages and limitations. As this field continues to burst, we believe new methods for m^6^A mapping will appear. For instance, although DART-seq is recently applied to single cells,^57^ a new method that could quantitatively map m^6^A at single-cell level would be extremely exciting. In addition, it is necessary to establish standard criteria for bioinformatic analysis in this field. Some of controversial findings might be attributed, at least some certain, to differences in the computational strategies used by different studies. Recent analysis of published MeRIP-seq datasets show that, because of high rates of background signals, only about 30% to 60% of m^6^A peak were reproducible. To improve the reliability in characterizing RNA modification, in vitro transcribed RNA product is introduced as negative control to reduce the false positive resulting from sequencing bias or RNA structure.^63^ Overall, each method established so far for m^6^A mapping definitely contributes to the fast advances of this field.

## 4. RNA m^6^A IN AML

**Figure 3.**
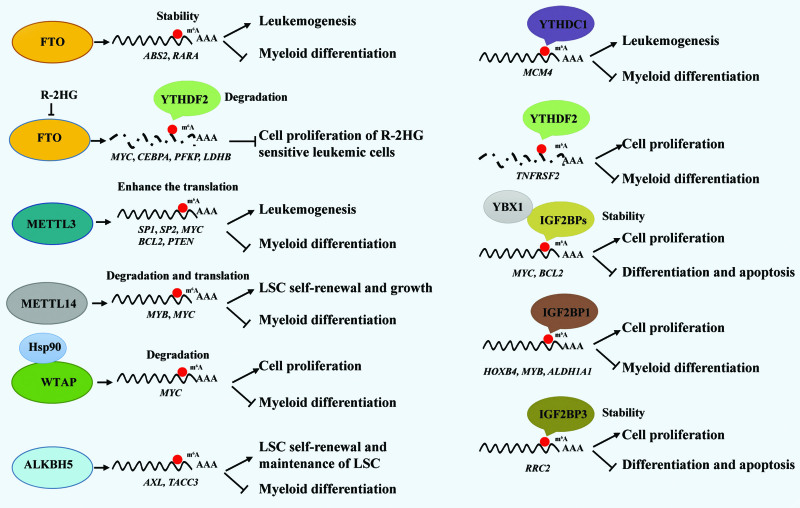
The roles and mechanisms of m^6^A modification in AML. m^6^A modification dysregulation can enhance AML leukemogenesis. m^6^A regulators act as oncogenes affects cell apoptosis, cell cycle, cell differentiation or LSC self-renewal via distinct mechanism. AML = acute myeloid leukemia, LSC = leukemia stem cell.

In recent years, the roles and molecular mechanisms of RNA m^6^A in different physiological and pathological conditions have been widely uncovered. Numerous studies have shown that methyltransferase complex components (METTL3, METTL14, WTAP), demethylases (FTO, ALKBH5), and the m^6^A binding protein (YTHDF2, YTHDC1, and IGF2BPs) are all highly expressed in AML. m^6^A regulators can function as oncogenes by targeting corresponding oncogenes, which are closely related to the occurrence, development, clinical treatment, and prognosis of AML. Hereby, we focus on AML and discuss current main advances in this field (Fig. 3).

### 4.1. Roles and mechanisms of m^6^A in AML

FTO is the first m^6^A modifier that is reported to play an oncogenic role in AML.^64^ High expression of FTO is observed in leukemia cells from different subtypes of AML. Knockout or inhibition of FTO could inhibit the self-renewal of LSCs, and impair the AML development. Mechanistically, FTO controls the degradation of ASB2 and RARA mRNA in an m^6^A-dependent manner.^64^ As FTO is a α-ketoglutarate-dependent dioxygenase, its activity can be competitively inhibited by R-2-hydroglutarate (R-2HG), which is structurally closed to α-ketoglutarate. Inhibiting FTO by R-2HG was found increasing the overall level of m^6^A without affecting FTO expression and decreasing the stability of MYC and CEBPA mRNA in R-2HG-sensitive leukemia cells.^65^ R-2HG also abrogates FTO/m^6^A/YTHDF2-mediated upregulation of 2 critical glycolytic genes *phosphofructokinase platelet (PFKP*) and *lactate dehydrogenase B (LDHB*), thereby suppressing the glycolysis of leukemia cells.^66^

Subsequently, the roles of METTL3 and METTL14 in AML were also revealed.^67–69^ METTL3 deletion inhibits cell growth, promotes differentiation and apoptosis, and significantly prolongs survival in murine AML xenografts. These effects are mainly mediated by altering translation of c-MYC, BCL2, and PTEN mRNA in an m^6^A-dependent manner.^67,68^ In addition, mechanistically, METTL3 alone could bind chromatin and localize to transcriptional start site (TSS) of active genes. Promoter-bound METTL3 recruits CEBPZ and regulates the translation of downstream oncogenic drivers SP1 and SP2, subsequently regulating the expression of c-MYC.^68^ METTL14 exerts its oncogenic role by regulating MYB and MYC mRNA degradation and translation through m^6^A modification, and also plays a critical role in AML development and maintenance.^69^ WTAP was found overexpressed in AML patients, which are inversely correlated with overall survival in patients with AML. Depletion of WTAP suppresses cell proliferation, cell cycle, differentiation, and colony formation in AML cells. WTAP has an important role in AML oncogenesis, which is a novel client protein of Hsp90.^70^ Thus, these works provide rationale for FTO/METTL3/METTL14/WTAP as potential therapeutic targets for AML treatment.

Recently, we reported that ALKBH5 is required for the development and maintenance of AML and self-renewal of LSCs but not essential for normal hematopoiesis. Mechanistically, KDM4C regulates ALKBH5 expression via increasing chromatin accessibility of ALKBH5 locus, by reducing H3K9me3 level and promoting recruitment of MYB and Pol II. Moreover, ALKBH5 affects mRNA stability of receptor tyrosine kinase AXL in an m^6^A-dependent way.^71^ Complementary findings also identified the crucial role of another ALKBH5 for the self-renewal and maintenance of LSCs in AML. They found that ALKBH5 may exert tumor-promoting effects in AML by post-transcriptional regulation of a critical target, TACC3, a prognosis-associated oncogene in various cancers.^72^ Therefore, these 2 studies clearly uncover the selective and important role of ALKBH5 in the pathogenesis of AML.

The roles of m^6^A readers or related factors in leukemogenesis are also being recognized. Knockout of YTHDC1 severely impedes the development and maintenance of AML as well as LSC self-renewal in mice. Mechanistically, YTHDC1 regulates leukemogenesis through MCM4, which is a critical regulator of DNA replication.^73^ YTHDF2 deletion extends the half-life of m^6^A-modified transcripts including Tnfrsf2 to selectively compromise AML initiation and propagation without harming normal hematopoiesis.^74^ Recently, we reported that YBX1 interacts with IGF2BPs to stabilize m^6^A-modified transcripts, including MYC and BCL2. Upon YBX1 loss, MYC and BCL2 undergo accelerated decay, thus compromising AML cells.^48^ The role of IGF2BPs in leukemia is also being uncovered recently. Higher expression of IGF2BP2 predicts poor prognosis in AML patients,^75^ implying an important role of IGF2BP2 in AML, which need to be investigated. Deletion of IGF2BP1 affects proliferation, promotes myeloid differentiation, and decreases tumorigenic potential of AML cells by regulating HOXB4, MYB, and ALDH1A1.^76^ IGF2BP3 is also required for AML cell survival in an m^6^A-dependent manner, and IGF2BP3 loss significantly induces AML cell apoptosis, inhibits proliferation, and attenuates the ability of AML cells to develop leukemia in vitro and in vivo. Mechanistically, IGF2BP3 interacts with RCC2 mRNA and stabilizes the expression of modified m^6^A RNA.^47,77^ Taken together, these studies uncover the similar functional roles of different m^6^A modifiers (writers, erasers, and readers) via totally distinct mechanisms.

### 4.2. Identifying inhibitors of m^6^A modifiers for AML treatment

Studies discussed above have provide rationale for targeting some m^6^A modifiers as a potential therapeutic strategy in AML treatment, which drive seeking for their inhibitors. Several inhibitors targeting FTO and METTL3 are currently being identified. A selective small molecule inhibitor STM2457 of METTL3 is identified by a high throughput screening of 250,000 diverse drug-like compounds. STM2457 is able to bind to the SAM binding site of METTL3, which is structurally different from SAM and other known methyltransferase inhibitors. STM2457 does not recognize the homocysteine binding pocket used by SAM, but binds to the K513 site which is the known structural diversity of the cofactor-binding site of SAM-dependent methyltransferases. STM2457 also shows significant anti-leukemic effects in preclinical AML models.^48,78^ In addition, FB23 and FB23-2 is found to directly bind to FTO, and selectively inhibit FTO’s m^6^A demethylase activity. They also effectively inhibit AML progression in AML models.^79^ Although FB23-2 had a statistically significant effect on inhibiting the development of primary AML in mice, the inhibition rate for the compound was not satisfactory. Another 2 molecules, CS1 and CS2, have been screened out and identified as effective inhibitors of FTO, which can suppress its m^6^A demethylase activity by occupying the catalytic pocket and interfering with the binding of FTO with m^6^A-modified RNAs and then exert potent anti-leukemic efficacy in vivo and in vitro.^80^ The potent anti-tumor efficacy and minimal side effects of CS1 and CS2 suggest that they are highly feasible for clinical application. Thus, these findings highlight the promising potential for AML treatment by targeting of m^6^A modifiers. However, given the critical role of m^6^A modification under the physiological conditions, it is critical to carefully assess both the efficacy and safety of these inhibitors in the future.

## 5. CONCLUSION AND PERSPECTIVES

The biology of RNA modification has attracted tons of interests. It becomes clear that RNA RNA m^6^A plays critical roles in the pathogenesis of AML. Meanwhile, more and more very interesting scientific questions appear. For instance, given RNA m^6^A modification seems function as an adaptive-response system, how does this system sense and respond to various environments? How are the activities and expression of m^6^A modifiers regulated upon different responses? Our recent work found that KDM4C and MYB are upstream regulators of ALKBH5 in AML,^71^ it is of great interest to investigate how other m^6^A modifiers are dysregulated in leukemia in the future. Second, what determines the transcript target specificity of m^6^A modifiers? It remains unknown how the transcripts are selected by m^6^A modifiers. Our recent work implies that cofactors or RBPs might be recruited to regulatory machineries and play key roles in determining their target specificity. To be more exciting, developing better methods to comprehensively and quantitatively map m^6^A landscape in a single cell level will be necessary in the future. Finally, it is very hopeful that targeting RNA m^6^A modification would be a novel and attractive therapeutic approach for AML.

## ACKNOWLEDGMENTS

This work is supported by grants to H.Z. from Medical Science Advancement Program (Basic Medical Sciences) of Wuhan University (TFJC2018005) and from the Fundamental Research Funds for the Central Universities (2042021kf0225).

We acknowledge the members of our laboratory for helpful discussion.
